# Comparison of standard exponential and linear techniques to amplify small cDNA samples for microarrays

**DOI:** 10.1186/1471-2164-6-61

**Published:** 2005-05-04

**Authors:** Johan Wadenbäck, David H Clapham, Deborah Craig, Ronald Sederoff, Gary F Peter, Sara von Arnold, Ulrika Egertsdotter

**Affiliations:** 1 Department of Plant Biology and Forest Genetics, Swedish University of Agricultural Sciences, P.O. Box 7080, Uppsala, Sweden; 2 Department of Forestry, North Carolina State University, Raleigh, NC 27695, USA; 3 School of Forest Resources and Conservation, University of Florida, Gainesville, FL 32611, USA; 4 Department of Forestry, Virginia Polytechnic Institute and State University, Blacksburg, VA 24061, USA

## Abstract

**Background:**

The need to perform microarray experiments with small amounts of tissue has led to the development of several protocols for amplifying the target transcripts. The use of different amplification protocols could affect the comparability of microarray experiments.

**Results:**

Here we compare expression data from *Pinus taeda *cDNA microarrays using transcripts amplified either exponentially by PCR or linearly by T7 transcription. The amplified transcripts vary significantly in estimated length, GC content and expression depending on amplification technique. Amplification by T7 RNA polymerase gives transcripts with a greater range of lengths, greater estimated mean length, and greater variation of expression levels, but lower average GC content, than those from PCR amplification. For genes with significantly higher expression after T7 transcription than after PCR, the transcripts were 27% longer and had about 2 percentage units lower GC content. The correlation of expression intensities between technical repeats was high for both methods (R^2 ^= 0.98) whereas the correlation of expression intensities using the different methods was considerably lower (R^2 ^= 0.52). Correlation of expression intensities between amplified and unamplified transcripts were intermediate (R^2 ^= 0.68–0.77).

**Conclusion:**

Amplification with T7 transcription better reflects the variation of the unamplified transcriptome than PCR based methods owing to the better representation of long transcripts. If transcripts of particular interest are known to have high GC content and are of limited length, however, PCR-based methods may be preferable.

## Background

The analysis of transcript abundance in samples of total RNA using standard techniques such as northern blotting or microarrays requires microgram quantities of total RNA. In our experience, a microarray analysis incorporating a loop design and reciprocal labeling with Cy™3 and Cy™5 dyes, requires 80 micrograms of total RNA per sample [1]. It is often inconvenient or impossible to obtain sufficient quantities without an amplification step, particularly if tissue sections are to be analyzed. Exponential amplification of cDNA by a standard PCR procedure [2] may result in the differential amplification of particular transcripts, since sequences differ in the rate with which they can be amplified by PCR [3]. To minimize this problem, the sequences to be amplified can be limited to about 300 nucleotides at the 3'-terminus of the cDNA; this can be achieved by ultrasound treatment [4] or by limiting the concentration of deoxynucleotides in the PCR reaction mixture [5]. These methods are promising but not yet in standard use. An alternative approach is linear amplification by *in vitro *transcription from a strong promoter such as a T7 phage promoter [6]. Linear amplification has been shown to retain the relative frequencies of transcripts with reasonable fidelity over a wide amplification range [7-10]. Many aspects of the high efficiency and reliability of linear and exponential amplification methods have been studied earlier. These deal mainly with comparisons between unamplified and amplified material and indirectly between different amplification methods [11,12]. The distorting effect in mRNA abundance of linear and exponential amplification techniques in relation to the sequence and GC content of the genes has been hypothesized [12], but very little evidence has been put forward to support this in relation to the characteristics of individual transcripts.

Commercial kits are available for both exponential (Super SMART™ from BD Biosciences Clontech) and linear (Message Amp™ from Ambion) amplification. Here we compare expression levels determined with cDNA microarrays hybridized with cDNA obtained from thin sections of secondary xylem tissue from *Pinus taeda *amplified by these two different strategies, or after using unamplified cDNA for hybridization. We have addressed the questions: how well do the results agree with each other in a direct comparison? What are the characteristics of the sequences showing preferential amplification by exponential or linear amplification?

## Results and Discussion

### Comparison of unamplified- and amplified targets

A typical sample for amplified Super SMART™ PCR-product yields a distribution of sizes from 500 bp-6000 bp with a peak centered at 900 bp (Clontech, Super Smart PCR cDNA Synthesis Kit User manual). A typical sample for amplified Message Amp™ aRNA yields a distribution of sizes from 250 nt-5500 nt with a peak centered at 1000–1500 nt (Ambion, Catalog #1752). The distributions of our amplified material agree well with the manufacturers' data (See Additional file 1 and 2). It has been reported that PCR amplification requires less RNA, is more reproducible and generates better target transcripts than linear amplification [5,13], at least if the sequences are limited to the 3'- end. Linear T7 amplification has however been widely used when starting material is limiting. Recently some researchers have reported bias in their data. In some studies the bias is said to be of minor importance, systematic and reproducible, affecting all the samples in the same way and therefore potentially controllable in the normalization (e.g. to calculate fold change) [10,14]. In other studies the bias from different amplification protocol is affecting the general ratios of gene expression [5,12]. Part of the bias may arise from the T7 RNA polymerase's intrinsic nucleolytic activity that appears during extended incubation [15]. Other bias is maybe introduced owing to the characteristics of the individual transcripts.

We have found a preferential amplification of certain nucleotide sequences by the Super SMART™ PCR relative to a nonamplified target in earlier membrane array experiments, where the targets were prepared from the samples of lignified planings and nonlignified xylem scrapings (data not shown). The correlation (R^2 ^) between transcript abundance using unamplified and Super SMART™ PCR amplified targets was 0.77 for scrapings and 0.68 for planings.

Comparing five lines of *Picea abies *shoots where the first biological replicate consisted of unamplified targets and the second biological replicate consisted of targets amplified with T7 transcription we obtained a correlation of R^2 ^= 0.74 (data not shown). Ambion has reported R^2 ^= 0.87 [16] between technical repeats.

Plots of the individual gene transcript abundance of unamplified versus amplified target should give a straight line of slope 1 if the overall expression is preserved. However, there is some nonlinear behavior in both cases. For unamplified versus PCR amplified target the curve is generally nonlinear and lower abundance transcripts are under-represented and highly expressed transcripts are amplified better than average. For the unamplified versus T7 amplified targets a very small minority of highly expressed transcripts do not follow the linear slope of around 1.

For the comparison between unamplified and PCR amplified targets the 95% confidence intervals for the fold-changes were as follows: For unamplified material: Downregulation, 2.3–8.0; upregulation: 1.1–1.3. For PCR-amplified material: Downregulation, 1.3–1.7; upregulation, 1.0–3.0. The differences between unamplified and PCR amplified targets were statistically significant.

For the highly significant (p < 0.0001) differentially expressed genes between lines in each of the ten comparisons of unamplified and T7 amplified targets, the 95% confidence intervals for the fold-changes were as follows. For unamplified material: Downregulation, 1.5–3.2 (all), and 2.7–7.5 (top); upregulation: 1.3–2.8 (all), and 2.6–4.4 (top). For T7-amplified material: Downregulation, 1.0–2.7 (all), and 2.5–4.2 (top); upregulation: 1.2–3.0 (all), and 2.3–4.3 (top). The differences between unamplified and T7 amplified targets are generally not statistically significant although the fold change for the unamplified targets were greater than for the T7 amplified targets indicating that some small bias may still exist when using T7 amplified relative to unamplified targets, especially for highly expressed transcripts.

However, in many situations there is no possibilty of using unamplified targets and amplification is required. Thus, starting with small amounts of secondary xylem tissue we compared PCR and T7 RNA polymerase amplification methods directly to investigate if, and how, the biases differ from each other.

### Expression characteristics of transcripts amplified by PCR or T7 transcription

**Figure 1:**
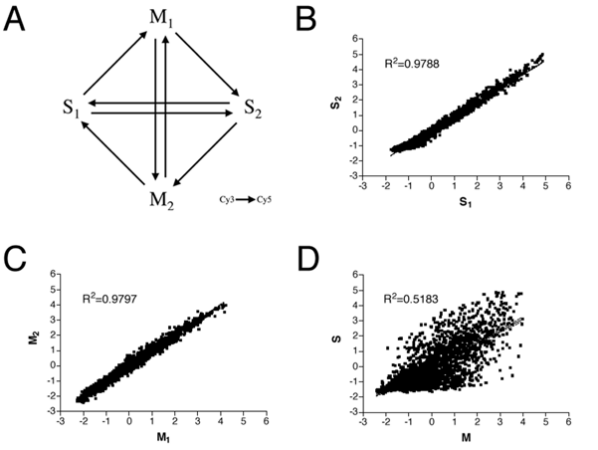
**Global comparison of PCR and T7 amplification techniques**. (A) Microarray experimental design. S_1 _and S_2_, and M_1 _and M_2 _are technical repeats of PCR (S) and T7 amplification (M) respectively. Each arrow represents one slide where the sample at the base of the arrow is labeled with Cy™3 and the sample at the tip of the arrow is labeled with Cy™5. (B-D) Correlation of results within and between amplification techniques; the values are least square means of expression of each of the genes represented on the array. (B) The correlation between two technical repeats of gene expression after S amplification. (C) The correlation between two technical repeats of gene expression after M amplification. (D) The correlation between gene expression after S and M amplification. Each amplification method produces highly consistent results (R^2 ^= 0.98) whereas the correlation of the results given by the two different methods is considerably lower (R^2 ^= 0.52) indicating bias in one or both amplification techniques.

The two methods of amplification were compared to each other four times and twice to themselves in a fully balanced flip dye experimental design including technical repeats (Figure 1A). Only few spots were flagged as bad and excluded from further analysis. The percentage of detectable spots (above background) on each array and in each channel was 88% using T7 amplification and 71% using PCR amplification. The percentage of saturated spots was around 1% in all cases.

After normalization the correlation of transcript abundance for each gene between technical repeats was very high, R^2 ^= 0.98, after both PCR- (Figure 1B) and T7 amplification (Figure 1C). In contrast, the correlation between the two different amplification methods for both technical repeats was considerably lower, R^2 ^= 0.52, (Figure 1D), indicating bias in one or both amplification techniques. As previously mentioned the correlation between unamplified and amplified transcript abundance was intermediate, indicating that both amplification methods have bias and that these biases are different from each other.

The genes present on the microarray were divided into two groups according to whether the PCR amplified transcripts (S') or the T7 amplified transcripts (M') were more abundant. The S' group was 9% larger than the M' group.

**Figure 2:**
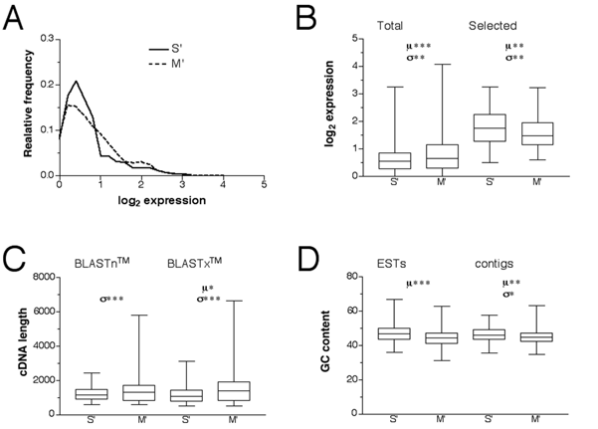
**Statistical analysis of microarray targets**. Characteristics of genes (represented by *Pinus taeda *ESTs) showing preferential amplification by one method or the other. From the results of the microarray normalization, the genes were divided into two groups, those showing higher expression for PCR amplified transcripts (S') and those showing higher expression for T7 amplified transcripts (M'). (A) Distribution of expression for all the genes (2190 ESTs) in the S' and M' groups. (B) Transcript abundance of all the genes and selected genes (represented by 309 ESTs) in the S' and M' group. (C) Transcript lengths for the two groups, estimated by finding the *Arabidopsis thaliana *homologs either from the nucleotide sequence (BLASTn™) or the amino acid sequence (BLASTx™) of the *Pinus taeda *contigs. The variance of the transcript length is significantly smaller for the S' group than for the M' group for both the nucleotide and protein estimates. There is furthermore a significantly greater mean length for the M' group than for the S' group. (D) GC content sequenced ends of the selected genes of the S' and M' groups. The S' group is significantly more GC rich than the M' group, for both ESTs and contigs. Bars indicate the range; boxes extend from the 25^th ^to the 75^th ^percentile, with a horizontal line at the median.

A relative frequency distribution plot of expression levels revealed a narrower peak for S' than for M' transcripts (Figure 2A). The arithmetic expression values showed a significantly greater mean for M' (1.76) than for S' (1.64) and a higher variance although the coefficient of variation was lower for M' (81.6%) than for S' (86.6%). The distribution of the data implies a broader population of transcript species present in the T7 amplified target.

Using the criteria for statistical significance described in methods, 309 ESTs (14%) showed different expression levels between the two amplification methods with 131 ESTs in the S' group and 178 ESTs in the M' group. The arithmetic mean of the S' group (3.40) was statistically higher than the M' group (2.95) and the S' group had higher variance (Figure 2B). The coefficient of variation was lower for M' (33.4%) than for S' (36.3%). The reason for the opposite trend observed for this subset of genes may reflect the differences in detectable spots and the amplification kinetics between PCR and T7 transcription.

### Transcript characteristics amplified by PCR or T7 transcription

As shown above, out of the genes (309 ESTs) showing statistically significant abundance differences between the amplification methods, 36% more were found in the M' group than in the S' group. One possibility for why 36% more were found in the M' group is that the complexity of the T7 amplified transcripts is greater. To assess this we analyzed the length of the sequences on the array. Previous analyses of protein sequences showed about half of *Pinus taeda *ESTs on the array have an apparent homolog in *Arabidopsis thaliana *(increasing with length up to 90%). For these ESTs the sequence similarity is typically distributed over the full length of the contig indicating a substantial conservation of genes between these two species, suggesting a common functional genome [17]. From the BLASTn™ (nucleotide level) and BLASTx™ (amino acid level) searches relating the contig data to *Arabidopsis thaliana *homologs, the corresponding *Pinus taeda *full-length cDNAs were estimated. The contig lengths constitute on average about 45 % of the total cDNA lengths spotted on the array. For both the nucleotide and the amino acid levels there was a highly significant 60% greater variance in length of the M' group than of the S' group. At the amino acid level there was a significant 26.9% greater mean length of the M' group (1580 bp) than the S' counterpart (1245 bp). The maximum length of transcript present was also considerably greater in the M' group than the S' group (Figure 2C). In contrast to the contigs the singleton ESTs in the S' group (482 bp) had a significantly greater mean sequence length than those in the M' group (428 bp). The reason for this discrepancy is unclear but could reflect a difference in efficiency of the sequencing polymerase resulting from difference in the amount of secondary structures in the sequences from the two sets. The M' group contained 60% of the ESTs and contigs with nucleotide and amino acid homology to *Arabidopsis thaliana *reflecting both an initially greater transcript population as well as differences in transcript lengths. In conclusion, the possibility of getting transcripts of greater length and larger variability is considerably higher when using T7 amplification rather than PCR amplification.

### Importance of GC content for amplification

Comparison of the selected genes (309 ESTs) differentially represented in the two amplification methods, the GC content of the ESTs, contigs and *Arabidopsis thaliana *cDNAs (on a nucleotide level) there was a significantly greater mean GC content for the sequences of the S' group than for those of the M' group. The difference was 2.7 percentage units for ESTs, and 1.4 percentage units for the corresponding contigs (Figure 2D). There was a similar difference for the cDNAs although only about 10% of the contigs were found to have a BLASTn™ score above 100 bits. Interestingly, for a smaller group of 80 contigs (40 from S' and 40 from M') showing the greatest fold changes between methods, the difference in GC content increased from 1.4 to 2.2 percentage units, due to an increase in GC content for the S' group. Additionally, the mean length of the 40 ESTs from the S' group (1428 bp) was significantly greater than the mean length of the 40 ESTs from the M' group (1275 bp). It appears that transcripts with a high GC content are amplified faster by PCR than by T7, often overriding the effect of length. If the GC content is nearer the average, long transcripts are favored by T7 amplification. The GC effect is presumably explained by the temperature of extension, which is 68–72°C for Taq polymerase and 37°C for T7 polymerase; high temperature favors polymerization through GC-rich areas. Evolution has in general tuned the cellular machinery, including polymerases, to fit the temperature environment of an organism. This might be reflected in the GC content and the temperature environment of the original organism for each polymerase. The GC content of a *Pinus *species genome is about 40%, which is considerably closer to the 48% GC content of T7 phage (or the 50% GC content of *Escherichia coli*, the typical host of T7 phage), than for the 67% GC content of *Thermus aquaticus *[18-20]. It implies that T7 transcription of the *Pinus taeda *transcriptome or consequently other transcriptomes with similar GC content in most cases is a better choice than PCR based techniques.

## Conclusion

In summary, the two main approaches to amplification of small amounts of RNA for microarray studies, PCR and T7 transcription both introduce bias compared to the unamplified target and the nature of the bias is different for each method. Our results show that amplification by T7 RNA polymerase gives transcripts with a greater range of lengths, greater estimated mean length, and greater variation of expression levels, but lower average GC content, than those from PCR amplification. Amplification with T7 transcription would therefore better reflect the variation of the unamplified *Pinus taeda *transcriptome and other comparable transcriptomes than PCR based methods. If transcripts of particular interest are known to have high GC content and are of limited size, however, PCR based methods may be preferable. The results demonstrate the need to pay attention to possible biases introduced by the amplification methods and that in certain projects different amplification techniques should be tested and optimized before routine use.

## Methods

### Target extraction and amplification

Polyadenylated RNA was extracted from individual 30 μm cryotome sections (3 mm × 3 mm) through the cambial region of *Pinus taeda *L. using Dynabeads (Dynal Biotech, Oslo, Norway). The mRNA samples were reverse-transcribed and the resulting cDNA were amplified by a) exponential PCR amplification by Super SMART™ (BD Biosciences Clontech, Palo Alto, CA, USA), or b) linear T7 amplification through Message Amp™ aRNA kit (Ambion, Austin, TX, USA). The Super SMART™ cDNA products were directly labeled by Klenow with Cy™3 or Cy™5 dUTPs (Amersham Biosciences, Piscataway, NJ, USA). The Message Amp™ aRNA products were reverse-transcribed with aminoallyl-modified dUTPs (Sigma, St. Louis, MO, USA) and labeled by coupling to free Cy™3 or Cy™5 dye (Amersham Biosciences) (Table 1).

**Table 1:** Flow chart of the exponential- and linear amplification techniques with Klenow- and aminoallyl labeling respectively

**Exponential Amplification with PCR DNA Polymerase**	**Linear Amplification with T7 RNA Polymerase**
	

### Microarray hybridization and probe selection

Microarray hybridization and stringency washes have been described previously [21,22]. cDNA microarrays based on 2190 *Pinus taeda *ESTs from the NSF unigene set (Forest Biotechnology Group, NCSU, NC, USA) [17] were hybridized with the labeled targets. The PCR and T7 amplification methods were compared in a fully balanced, flip dye design encompassing eight microarray slides (Figure 1A). The microarray data is MIAME compliant [GEO:GPL1880].

### Data normalization and analysis

The consistency of each method was assessed by dividing the samples into two technical repeats. The slides were scanned using a ScanArray^® ^4000 Microarray Analysis System (GSI Lumonics, Ottawa, Canada). Raw intensity values were collected with QuantArray^® ^software (GSI Lumonics) and spots were visually inspected for spot morphology and background. No background subtraction was applied because backgrounds were low and subtraction can introduce bias. The microarray intensity data was normalized using a mixed model system [21,23-25] in SAS/STAT Software version 8 (SAS Institute Inc., Cary, NC, USA). The log_2 _fold change in abundance was used to divide the selected genes in two groups depending on sign.

The normalized log_2 _fold change (essentially a ratio of the least square means of Super SMART™- and Message Amp™-amplified transcript abundance derived from the mixed model) with a probability value of p < 0.001 and array- and array*dye interaction variance lower than 0.001 were used to select genes with significant changes in abundance (represented by 309 ESTs). The absolute values (i.e. a rescaling of the data disregarding the sign) of the log_2 _fold change abundance were then used in the subsequent statistical analysis. The abbreviations used are: S = abundance of Super SMART™-amplified transcripts; M = abundance of Message Amp™-amplified transcripts; S'= [log_2_(M/S)], S>M; and M' = [log_2_(M/S)], S<M. In all the comparisons the individual transcripts are represented by ESTs.

The lengths of the cDNAs represented on the microarray were estimated based on full length *Arabidopsis thaliana *[26] homolog sequences using the *Pinus taeda *ESTs and contigs [27]. The top *Arabidopsis thaliana *homolog cDNAs with a score greater than 100 bits were selected for *Pinus taeda *ESTs or contigs on nucleotide level (using BLASTn™ and the AGI transcripts (-introns, +UTRs) dataset) or amino acid level (using BLASTx™ and the AGI proteins dataset).

All the *Pinus taeda *ESTs and contigs including those subsets showing homology to *Arabidopsis thaliana *cDNAs were then analyzed for sequence length, GC content as well as log_2 _fold change abundance.

The corresponding groups in each subset were analyzed with Prism Software version 3 (GraphPad Software Inc., San Diego, CA, USA). F-tests were used for evaluating a group's compliance with Gaussian distribution. When the normal criteria were met for two groups, one-way ANOVA analysis (with Bonferroni post test) and unpaired t-tests with or without applicable Welch's correction (not assuming equal variances) were performed. When the normal criteria were not met the nonparametric Mann-Whitney test was performed.

## Authors' contributions

JW, DHC, GFP and UE carried out the laboratory work. JW, DHC, DC, SvA and UE participated in the normalization and analysis of data. JW, DHC, DC, RS and UE conceived the study, and participated in its design. JW, DHC, RS, GFP, SvA and UE carried out the drafting of the manuscript. All authors read and approved the final manuscript.

## Supplementary Material

Additional File 1 Size distribution of Super SMART™ amplified cDNAs (1% agarose gel) Click here for file

Additional File 2 Size distribution of Message Amp™ amplified aRNAs (Electropherogram, LabChip) Click here for file
