# Supplementary figures and images for: Comparison of standard exponential and linear techniques to amplify small cDNA samples for microarrays
Source: BMC Genomics. 2005 May 4;6:61. doi: 10.1186/1471-2164-6-61 (PMC1134654; doi:10.1186/1471-2164-6-61)

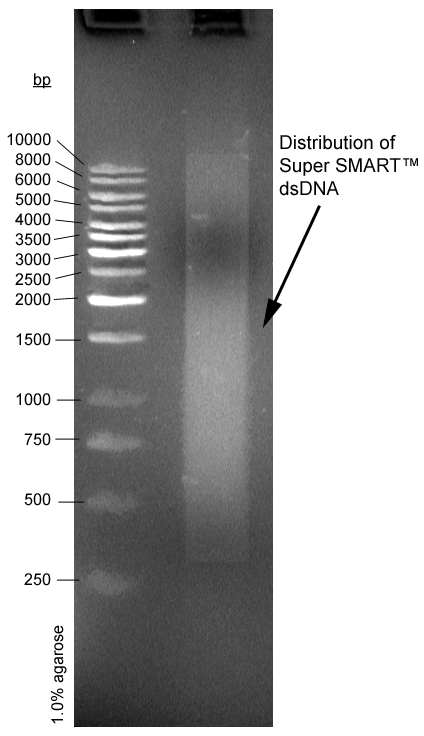

Supplement: Additional File 1 — Size distribution of Super SMART™ amplified cDNAs (1% agarose gel) [file 1471-2164-6-61-S1.tiff]

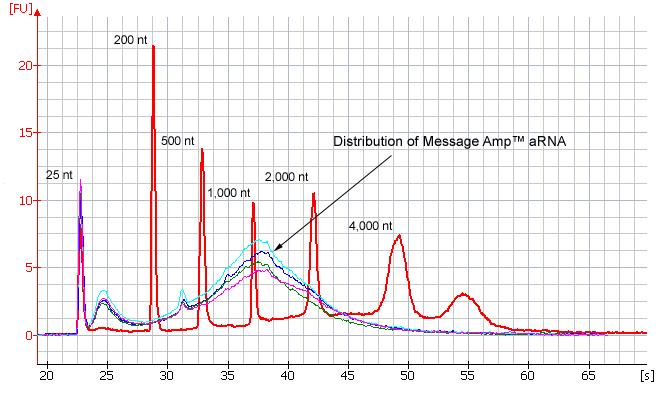

Supplement: Additional File 2 — Size distribution of Message Amp™ amplified aRNAs (Electropherogram, LabChip) [file 1471-2164-6-61-S2.tiff]
